# Effect of curcumin and three analogues on pre-osteoblast cells’ viability, differentiation, and gene expression

**DOI:** 10.1590/1807-3107bor-2024.vol38.0123

**Published:** 2024-12-09

**Authors:** Ana Flor Sá, Ivana Márcia Alves Diniz, Renata Barbosa de Oliveira, Marina Gonçalves Diniz, Maria Esperanza Cortés, Letícia Lopes de Souza, Carlos Delfin Chávez Olórtegui, Frederico Santos Lages

**Affiliations:** (a) Universidade Federal de Minas Gerais – UFMG, School of Dentistry, Department of Restorative Dentistry, Belo Horizonte, MG, Brazil.; (b) Universidade Federal de Minas Gerais – UFMG, School of Pharmacy, Department of Pharmaceutical Products, Belo Horizonte, MG, Brazil.; (c) Universidade Federal de Minas Gerais – UFMG, Department of Pathology, Biological Sciences Institute, Belo Horizonte, MG, Brazil.; (d) Universidade Federal de Minas Gerais – UFMG, Department of Biochemistry and Immunology, Biological Sciences Institute, Belo Horizonte, MG, Brazil.

**Keywords:** Curcumin, Cell Differentiation, Cell Survival, Osteoblasts

## Abstract

Curcumin, found in turmeric rhizomes (*Curcuma longa L.*), has been widely studied for its potential health benefits, including anti-inflammatory, antioxidant, and wound-healing properties. However, due to its low bioavailability and unfavorable pharmacokinetics, analogous compounds have been developed to obtain better biopharmaceutical characteristics and enhanced biological effects. In this study, we evaluated the activity of curcumin and three of its synthetic analogues (DMAD, DMAM, and RI75) on the viability and differentiation of a pre-osteoblastic cell line (MC3T3-E1). We also assessed the expression of key genes involved in tissue regeneration: vascular endothelial growth factor (*vegf*), stromal-derived growth factor 1 (*SDF-1/CXCL12*), and runt-related transcription factor 2 (*runx2*). The cells were treated with curcumin and the three analogues at concentrations of 10, 30, or 50 μM. All tested analogues and curcumin exhibited moderate to no cell toxicity compared to the cells treated under standard conditions across all concentrations after 24, 48, and 72 hours. Only the RI75 analogue showed upregulation of *SDF-1,* a crucial factor in tissue regeneration. Compared to curcumin, the DMAM and RI75 analogues also upregulated *runx2* and *vegf*, both associated with osteodifferentiation. The RI75 analogue demonstrated greater mineralization than curcumin, and both promoted more nodule formation than the untreated control. Our data suggest that the curcumin analogue RI75 at 50 μM presents similar toxicity but enhanced biological activity compared to natural curcumin, making it a promising substance for material biomodifications.

## Introduction

Natural products are vital sources for new drug discoveries.^1^ Curcumin (CUR), the primarynatural polyphenol extracted from the turmeric (*Curcuma longa*) rhizome, is a medicinal plant that has been used for centuries in traditional oriental medicine. The biological activities of curcumin have been continuously studied over the years.^2–4^ Studies have demonstrated that CUR possesses anti-inflammatory, wound-healing, antioxidant, antimicrobial, and anticancer properties.^3–5^.

CUR has shown a multitude of therapeutic benefits in various pathological conditions, including oral diseases such as oral mucosal diseases, oral cancer, and periodontal diseases.^3,5–9^ However, despite its popularity and potent biological activities, CUR's clinical efficacy is limited by several factors, including poor water solubility, limited physicochemical stability, low oral bioavailability, rapid metabolism, and a short biological half-life.^5,10–12^

To address these limitations, ongoing efforts have focused on developing synthetic curcumin analogues with improved pharmacological properties.^7,11^ Different curcumin analogues inhibit inflammation and bone resorption in different experimental models, and they have demonstrated the ability to reduce inflammatory cell infiltration and bone resorption in LPS-induced models of periodontitis.^7,11–14^ In a previous *in vitro* study, Son, Kim, and Jang (2017) demonstrated that two curcumin analogues enhanced osteoblast differentiation in both bone marrow mesenchymal cells (C3H10T1/2) and pre-osteoblastic cells (MC3T3-E1), as well as increased the expression of osteogenic *runx2* gene.^15^ The published results, so far, provide strong support for the continued investigation of curcumin analogues as potential drug candidates, reinforcing the hypothesis that structural molecular changes can improve the pharmacological properties of natural curcumin.

**Figure 1:**
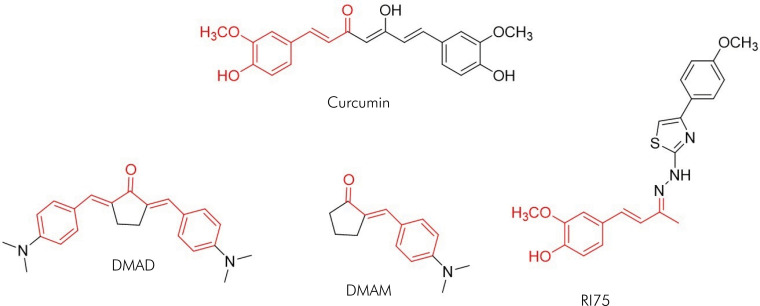
Comparison of the chemical structures of curcumin and its analogues DMAD, DMAM, and RI75, with the similar moiety (α,β-unsaturated system) highlighted in red.

This study investigated curcumin analogues of mono- and bis-(arylmethylidene) cycloalkanones (Figure 1), specifically DMAD, DMAM and RI75, which were synthesized as described by Braga et al.^16^ and Lino et al.^17^ Currently, limited information is available regarding their biological effects. In 2014, Braga et al.^16^ investigated the antiparasitic activity of DMAD and DMAM at 100 μM, finding no antiprotozoal activities against *Trypanosoma cruzi* and *Leishmania* sp. In the study of Lino et al.,^17^ RI75 was tested for its antifungal activity and the results revealed some inhibitory effect on six different species. More recently, Costa et al.^18^ reported that RI75 exhibited an anti-inflammatory profile in mice, attenuating mechanical allodynia and paw edema induced by carrageenan. Collectively, the existing results encourage further investigation into the biological effects of these curcumin analogues. Moreover, these analogues are easily synthesized and can be obtained from low-cost and accessible materials.^18^

Understanding osteoblast biology, a crucial component of the bone environment, as well as the impact of drugs and biomaterials to promote bone repair, is essential for developing new therapies for bone reconstruction. This is particularly important for treating several dental and medical conditions. As the effects of DMAM, DMAD, and RI75 on osteoblastic biology remain largely unknown, this study was conducted to evaluate and compare their impact on cell viability and differentiation, including the assessment of pro-resolutive gene expression in the MC3T3-E1 cell line.

## Methodos

### Cell culture

MC3T3-E1 (ATCC Subclone 4 CRL-2593) pre-osteoblastic cells were cultured in a regular medium (RM) consisting of high-glucose Dulbecco's Modified Eagle's Medium (DMEM) (Sigma-Aldrich, St. Louis, USA), supplemented with 10% fetal bovine serum (FBS) (Gibco, ThermoFisher, Waltham, USA), and 1% penicillin-streptomycin (10,000 U/ml) (GIBCO/Life Technologies, Carlsbad, USA). For the cell viability assays, cells were seeded at a 3 x10^3^ cells/well density in 96-well microplates. For the mineralization assay, cells were seeded at a 2.5 x 10^4^ cells/well density in 24-well microplates. For the quantitative reverse transcription polymerase chain reaction (qRT-PCR) assay, cells were seeded at a 5x10^5^ cells/well density in 6-well microplates. The cells were incubated in a humidified atmosphere at 37°C with 5% CO_2_. A cell sub-confluence > 85% was considered adequate to perform the proposed assays.

### Drugs

DMAD, DMAM, and RI75 (Table 1) are curcumin analogues synthesized according to previously reported procedures.^16,17^ Curcumin and dimethylsulfoxide (DMSO) (both from Sigma-Aldrich, St. Louis, USA) were also used in this study.

**Table 1:** Name, structure, and molar mass (MM) of curcumin and curcumin analogues synthesized in the Chemical Pharmaceutical Laboratory of UFMG.

Name	Chemical structure	MM
DMAD	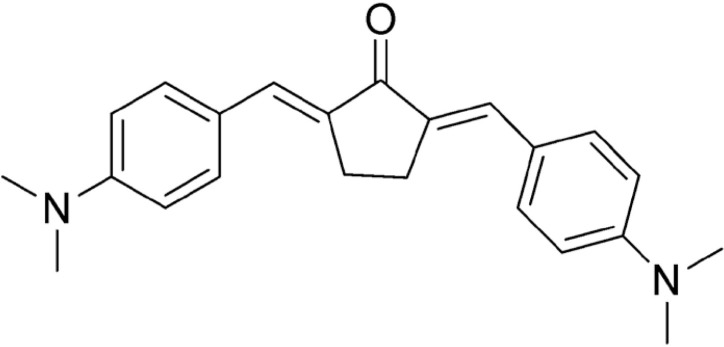	346 g/mol
DMAM	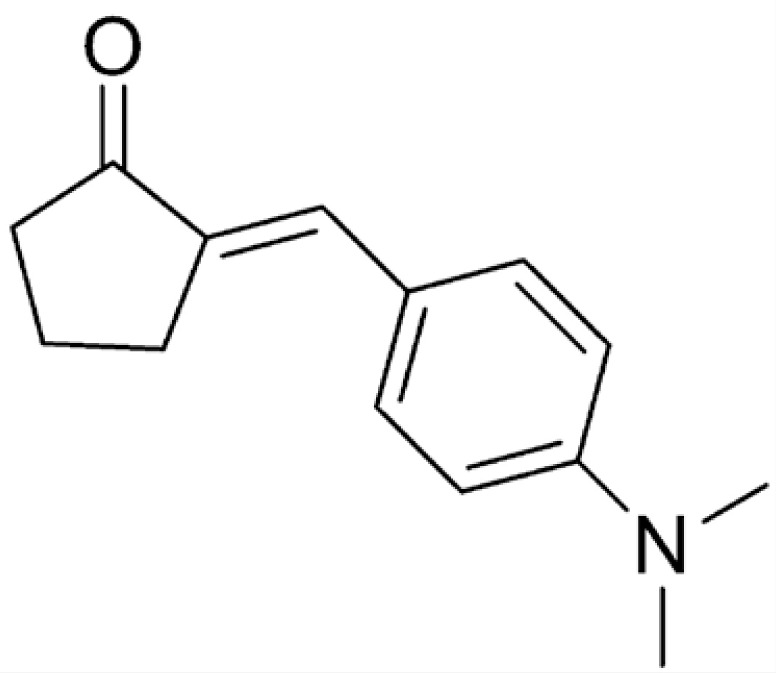	215 g/mol
RI75	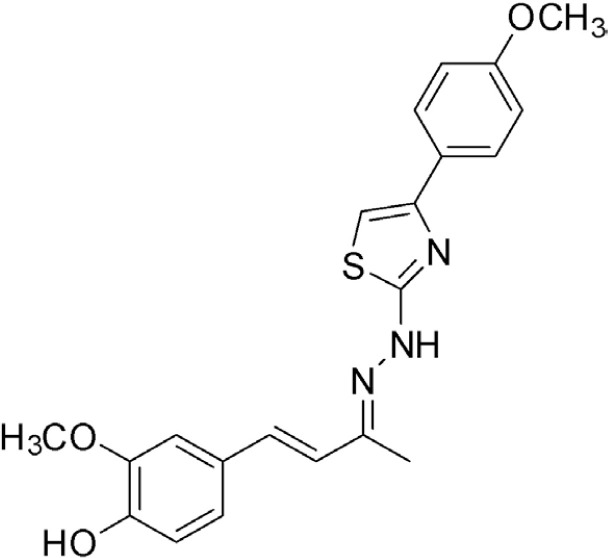	395 g/mol
CUR	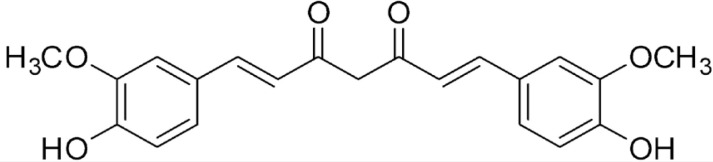	368 g/mol

Suspensions of CUR, RI75, DMAD, or DMAM were prepared by dissolving each compound in DMSO 0.4% volume/volume (v/v) and then diluting them in regular medium (RM). RM served as the untreated control group (MC) in all assays. The compounds (CUR, RI75, DMAD, or DMAM) were tested at concentrations of 10, 30, and 50 μM on MC3T3-E1 cell viability to determine the working concentration with a maximum of 50% toxicity value (IC50).^19^ The IC50 is a key parameter in drug discovery and pharmacology studies, indicating the potency of a drug; a lower IC50 value suggests that the drug is effective at low concentrations^20^. Based on these findings, gene expression and differentiation assays were conducted using the substances at 50 μM.

### Cell viability

Cell viability was measured in quadruplicate using the 3-(4,5-dimethylthiazol-2yl)-2,5-diphenyl tetrazolium bromide (MTT) assay (Gibco, Thermo Fisher, Waltham, USA). MC3T3-E1 cells were seeded for 24 hours before being treated with CUR, DMAD, DMAM, or RI75 at concentrations of 10, 30, or 50 μM for 24, 48, and 72 hours. Following treatments, cells were incubated with a 5mg/mL MTT solution according to the manufacturer's instructions. The absorbance of the formazan crystals, dissolved in DMSO, was measured at 540 nm using a Thermo Scientific Multiskan Spectrum MCC/340 spectrophotometer.

### Quantitative reverse transcription polymerase chain reaction (qRT-PCR)

The expression of the *runx2, vegf,* and *sdf-1 genes* in MC3T3-E1 cells was assessed by qRT-PCR after 10 days of cell stimulation. Total RNA was extracted from homogenized cells using silica-membrane spin columns (RNeasy Mini Kit, Qiagen, Valencia, USA). Single-stranded cDNA was synthesized from 250 ng of total RNA using the Super Script^®^ III cDNA synthesis kit (Life Technologies, Invitrogen) according to the manufacturer's instructions. Primer sets efficiency was confirmed prior to the experiments. Gene expression data were normalized relative to the housekeeping gene (*gapdh*). The primers used are listed in Table 2. qRT-PCT was performed using the SYBR^®^Green master mix (Life Technologies/Applied Biosystems) with the following cycling parameters: 50°C for 2 minutes, 95°C for 2 minutes, and 40 cycles of 95°C for 15 seconds and 60°C for 60 seconds.

**Table 2:** Specific primers for PCR.

Primer	Sequences (5’ → 3’)	
CXCL12	Forward	5’ -CAGTGACGGTAAACCAGTCAGC - 3’
	Reverse	5’ -TGGCGATGTGGCTCTCG - 3’
RUNX2	Forward	5’ - CAGGCAGGTGCTTCAGAACT - 3’
	Reverse	5’ - GGGGTGTAGGTAAAGGTGGC - 3’
VEGF	Forward	5’ - TGAACTTTCTGCTCTCTTGGGT - 3’
	Reverse	5’ - CCTGGGACCACTTGGCAT - 3’
GADPH	Forward	5’ - ACGGCCGCATCTTCTTGTGCA - 3’
	Reverse	5’ - CGCCAAATCCGTTCACACCGA - 3’

### Spontaneous mineralization

To evaluate the groups’ ability to induce spontaneous mineralization, an Alizarin Red S staining (Sigma–Aldrich) assay (AZR) was conducted. Cells were seeded and, after 24 hours, stimulated with CUR, DMAD, DMAM, or RI75 analogues at 50 μM concentration. Only RM was used in this assay to assess the spontaneous formation of mineralized nodules in the presence of CUR and its analogues. Mineralized nodule formation was assessed after 14 days. The cultures were fixed in 70% isopropanol, washed with PBS (3x), and the mineralized extracellular matrix was stained with 1% Alizarin Red S, pH 4.2, for 45 minutes. The cell cultures were then washed three times with PBS before documentation. For quantitative analysis, the nodules were solubilized by adding a solution of 10% acetic acid and methanol (4:1 v/v) to the wells for 30 minutes under agitation. The absorbance was measured at 490 nm using the Cytation 5 Cell Imaging Multi-Mode Reader (Biotek II). Data were normalized by analyzing representative images of each well, adjusting a threshold to quantify mean grey values using the Fiji Software (NIH).

### Statistical analysis

Data were analyzed using GraphPad Prism 10 software (GraphPad, San Diego, USA). The Shapiro-Wilk test was used to assess normality for all experiments. Statistical significance among groups was calculated using one-way ANOVA followed by Dunnet's test or Student *t* test. Differences were considered significant when p < 0.05 in all experiments. Data are expressed as means ± standard error.

## Results

### Cell viability (MTT)

**Figure 2:**
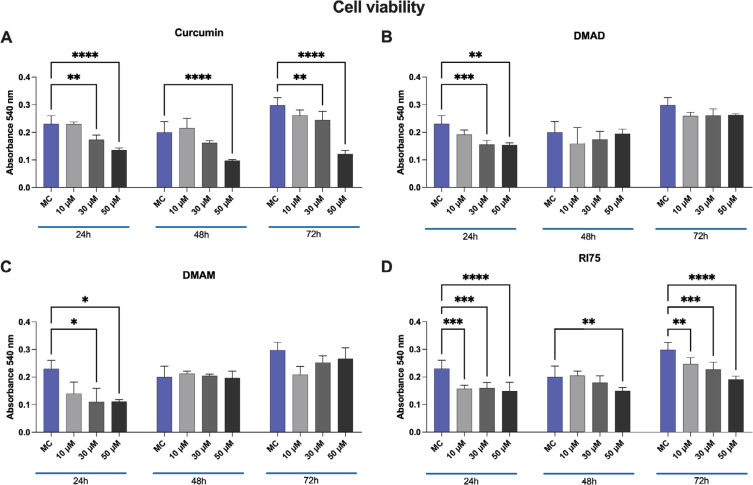
MC3T3-E1 cell viability after treatment with CUR (A), DMAD (B), DMAM (C), or RI75 (D).

All tested concentrations of CUR, DMAD, DMAM, and RI75 showed moderate or no cell toxicity compared to control cells cultured under ideal conditions (Figure 2). Except for RI75, all drugs exhibited a dose-dependent decrease in cell viability at the 24-hour time point, with statistical differences observed at 30 μM and 50 μM concentrations. At 24 hours, the RI75 analogue significantly decreased cell viability regardless of the concentration tested. At 48 and 72 hours, DMAD and DMAM showed no cytotoxicity, suggesting a short-lived biological influence of these compounds. The RI75 and CUR displayed similar cellular responses, with decreased viability at 48 and 72 hours, particularly at 50 μM. At this concentration, the viability of all analogues was either superior to or not statistically different from that of CUR across all time points (Figure 2). The 50 μM concentration was selected for subsequent bioassays since its cytotoxicity remained at moderate levels for the analogues, *i.e*., generally above the IC50 values.

MC: untreated control group. *p < 0.05; **p < 0.01; ***p < 0.001; ****p < 0.0001. Data are represented as mean ± SEM.

Results from the MTT assay are presented for 24-hour, 48-hour, and 72-hour intervals, in which MC3T3-E1 cells were treated with 10, 35, or 50 μM concentrations of CUR, DMAD, DMAM, or RI75. The results for CUR and the analogues at each concentration were compared with each other and with the untreated control group (MC) at each time interval.

**Figure 3:**
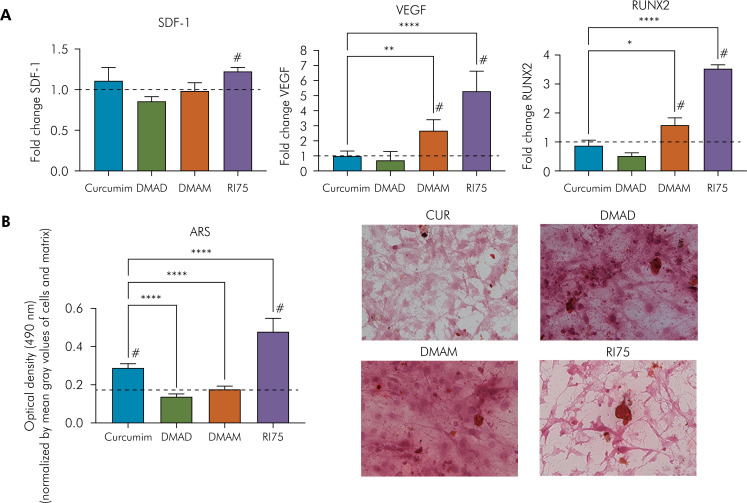
(A) Relative mRNA expression of sdf-1, vegf, and runx2 in MC3T3-E1 cells after treatment with 50 μM CUR, DMAD, DMAM, or RI75. (B) Bar graphs illustrating the osteoinductive potential of 50 μM CUR, DMAD, DMAM, or RI75 on MC3T3-E1 cells. Quantitative results of alizarin red S (ARS). (C): Representative bright-field images of ARS-stained MC3T3-E1 cells.

The pro-resolutive gene expression profile after 10 days of culturing is depicted in Figure 3A. No significant differences in *sdf-1* expression were observed among the tested drugs (Figure 3 A). However, RI75 showed an upregulation of *sdf-1* compared to the untreated control group. Both DMAM and RI75 – more notably – showed increased expression of *vegf* and *runx2* genes compared to CUR and the untreated control groups.

MC: Medium Control, untreated cells. #statistical difference compared to the untreated control. The dotted lines represent baseline values for the medium control/untreated group. Student t-test. *p < 0.05; **p < 0.01; ***p < 0.001; 1807-3107-bor-38-e123p < 0.0001; ANOVA followed by Dunnet's. Data are represented as mean ± SEM.

### Spontaneous mineralization

When normalized by the number of cells and the extracellular matrix present in the wells (mean gray values), quantification of mineral nodule solubilization revealed greater osteoinductive potential for CUR and its RI75 analogue compared with the untreated control group (Figure 3B). In contrast, DMAM and DMAD produced similar levels of matrix mineralization to the untreated control group and significantly less than CUR. Although images from the CUR and RI75 group qualitatively showed fewer stained nodules, the proportion of solubilized nodule optical density relative to the amount of available biological components indicated an increased mineral production in those samples (Figure 3B).

## Discussion

Curcumin (CUR) is a natural plant extract known for its high safety profile and low toxicity, offering numerous therapeutic benefits across various pathological conditions, including oral diseases such as oral mucosal diseases, oral cancer, and periodontal diseases.^2,6-9,13,21^ Studies on the effect of CUR on MC3T3-E1 cells have demonstrated its ability to enhance cell survival, migration, proliferation, and differentiation.^22,23^ However, due to CUR's pharmacological limitations, curcumin analogues have been continuously studied to achieve similar biological activities with improved pharmacological properties.^7,12,14–15,18^ DMAD, DMAM, and RI75 are newly synthesized curcumin analogues that have not yet been tested for their effects on osteoblastic cells. In this study, the effects of these analogues were investigated and compared to the effects of CUR on cell viability, gene expression, and spontaneous mineralization. The results indicate that these analogues exhibit moderate to no cytotoxicity, with RI75 outperforming CUR by promoting *sdf-1, runx2,* and *vegf* mRNA expressions and enhancing osteodifferentiation.

The cell viability results for CUR are consistent with published studies, which suggest a dose-dependent *in vitro* cytotoxicity.^22,23^ In the MTT assay, CUR exhibited no cytotoxic activity at concentrations of 10 μM, aligning with existing literature^23^. As expected, CUR reduced cell viability at concentrations of 30 μM and 50 μM. Previous reports have shown that 12.5-25 μM CUR induces osteoblast apoptosis, while doses greater than 50 μM cause osteoblast necrosis.^23^ In this study, CUR and its analogues exhibited moderate to no cytotoxicity across all tested concentrations. To assess the other effects on cell functions at the maximum potency of these compounds, further molecular and staining assays were conducted using CUR and its analogues at a concentration of 50 μM.

Overall, *sdf-1, vegf* and *runx2* are critical genes for wound healing and tissue repair.^24–31^ *Sdf-1* activity is tightly regulated due to its role in various homeostatic processes and inflammatory control. It acts as a key mesenchymal stem cell (MSC)-derived paracrine factor, facilitating wound healing and enhancing MSC paracrine signaling.^27^ Additionally, sdf-1 plays a significant role in bone growth and remodeling by recruiting osteoblast precursors.^25,27,28^ *Vegf* is one of the most important growth factors for regulating vascular development and plays a fundamental role in bone repair, given the close relationship between angiogenesis and osteogenesis.^26,29^, Vegf has also been reported to regulate osteoblast survival, enhance osteoblast differentiation, and facilitate bone formation during the bone healing process.^30^ *Runx2* is essential for osteoblast differentiation, with low expression in uncommitted mesenchymal cells and upregulated in pre-osteoblasts, peaking in immature osteoblasts.^31,32^ Moreover, a higher concentration of *vegf* in the medium has been shown to upregulate *runx2* expression in MC3T3-E1 cells.^33^

RI75 was the only analogue capable of promoting the upregulation of all three genes studied and demonstrated the greatest osteodifferentiation potential. DMAM also increased *vegf* and *runx2 gene expression*, although mineral nodule formation remained similar to that of the untreated control group. Conversely, CUR exhibited mineral formation at 14 days despite no upregulation of the target genes.

Evidence suggests that *runx2* protein is highly detected in immature osteoblasts and decreases as osteoblastic differentiation progresses.^32^ Although preliminary, our data suggest that *runx2* expression in the CUR (50 μM) group may have occurred prematurely and was no longer detectable by day 10. This could explain the lack of *runx2* expression and significant mineral nodule formation in the CUR samples. In contrast, the continuous stimulation of MC3T3-E1 cells by RI75, likely due to its greater bioactivity and bioavailability, may have led to prolonged upregulation of early osteogenesis markers. Interestingly, DMAM showed upregulation of *vegf* and *runx2* without significant mineral nodule formation. This may indicate a delayed mineralization response of MC3T3-E1 cells to DMAM, suggesting that increasing the concentration and/or duration of exposure could enhance bioactivity with less cytotoxicity.

It is important to highlight that the mineralization assay was conducted without the use of an osteogenic supplemented medium. Therefore, the results reflect spontaneous mineralization, a consequence of the MC3T3-E1 lineage's inherent commitment to the osteogenic pathway, rather than induced osteogenisis. MC3T3-E1 cells initially enter a proliferative phase characterized by minimal expression of differentiation markers, such as *runx2*, and the early synthesis of a collagenous extracellular matrix.^32^ Collagen matrix synthesis is crucial for the induction of differentiation markers, making ascorbic acid – an essential cofactor in collagen synthesis – also important for osteoblast differentiation.^34,35^ Several days after the initial induction of osteoblast marker genes, matrix mineralization begins, involving the growth of minerals within the matrix.^33,36^ However, mineral formation requires elevated levels of calcium phosphate ion products, which can be achieved by adding organic phosphates like β-glycerol phosphate to the medium.^37^ Therefore, if the medium had been supplemented with osteogenic factors, it is likely that mature osteoblasts would have deposited a more mineralized matrix.

Altogether, our data suggest that RI75 has the potential to induce terminal osteoblast differentiation by increasing runx2 expression and promoting vascularization through upregulation of the angiogenic signaling gene *vegf*. Notably, only RI75 demonstrated significant changes in osteogenic gene expression and corresponding protein levels. Thus, the promising osteogenic activity observed with the other analogues warrants further investigation in future studies.

In this study, the biological effects of CUR and its analogues were tested on MC3T3-E1 cell monolayers, which serve as a model for osteoblast biology, a critical component of the bone environment. The spontaneously immortalized murine calvaria cell line was selected because of its inherent commitment to the osteogenic lineage.^37^ However, it is important to note that at the transcriptional level, commercially available MC3T3-E1 subclones show little resemblance to primary murine calvaria pre-osteoblastic cells. This discrepancy means that data derived from these cells should be interpreted with caution.^37^ Furthermore, protein quantification was not performed to normalize mineral formation relative to extracellular matrix production. While our *in vitro* design has limitations in terms of clinical translation, the topical application of RI75 analogue could potentially influence the bone regeneration process. Based on our results, the analogues studied here show potential for bone regeneration. However, although the influence of curcumin analogues like RI75 on osteoblast differentiation appears promising, further confirmatory studies are necessary.

## Conclusion

Our study demonstrates that CUR analogues exhibit moderate to no toxicity and enhanced biological activity in MC3T3-E1 pre-osteoblastic cells. The findings suggest that the curcumin analogue RI75 has significant potential to induce terminal osteoblast differentiation by increasing the expression of the osteogenic-related genes *sdf-1*, *runx2,* and *vegf*. Additionally, the RI75 synthetic analogue shows a higher osteoinductive potential compared to CUR. Overall, these results encourage further investigation into curcumin analogues as promising drug candidates for biomaterial modifications aimed at tissue repair.
